# How does interactive virtual reality enhance learning outcomes *via* emotional experiences? A structural equation modeling approach

**DOI:** 10.3389/fpsyg.2022.1081372

**Published:** 2023-01-06

**Authors:** Hairu Yang, Minghan Cai, Yongfeng Diao, Rui Liu, Ling Liu, Qianchen Xiang

**Affiliations:** Department of Educational Technology, Institute of Education, China West Normal University, Nanchong, China

**Keywords:** interactive virtual reality, technical characteristics, learning experience, COVID-19 pandemic, CVTAE, presence, enjoyment, structural equation modeling

## Abstract

**Introduction:**

Interest in interactive virtual reality (IVR) is increasing due to its potential for embodied learning and group-led teaching. However, few studies have investigated the internal mechanism by which IVR technology features and learning experiences affect learning outcomes in terms of psychological and emotional value. Based on media technology models and the control value theory of achievement emotions (CVTAE), this study uses structural equation modeling (SEM) to investigate the correlations among the internal elements of IVR technology features, learning experiences, and learning outcomes. It also emphasizes the role played by emotional experience in this context.

**Methods:**

The sample referenced by this study consisted of 480 college students (193 males) who were simultaneously engaged in guided inquiry and learning in an IVR-based COVID-19 pandemic science museum in groups of 10.

**Results:**

The findings suggest that presence and perceived enjoyment have a key mediating effect on the relationship between virtual reality (VR) features and perceived learning outcomes in an IVR-based learning simulation. In addition, the results indicate that presence is more strongly correlated with perceived learning effects, while enjoyment is more strongly correlated with learning satisfaction.

**Discussion:**

These findings provide intellectual support and theoretical backing for VR-based instructional design and environmental development. Moreover, this study has practical value with regard to the future large-scale application of IVR to experiential teaching, group-led teaching, and the promotion of the digital transformation and intelligence upgrading in education.

## 1. Introduction

In recent years, the education sector has experienced a dramatic increase in the use of virtual reality (VR) to shape teaching experiences, as the Metaverse offers a fresh perspective on educational technology (Díaz et al., 2020; Rospigliosi, 2022). VR has been highly valued as an important technological innovation for future learning and teaching in recent EDUCAUSE Horizon Reports (Alexander et al., 2019; Brown et al., 2020). According to Mikropoulos and Natsis (2011, p. 769), VR can be defined as “a mosaic of technologies that support the creation of synthetic, highly interactive, three-dimensional (3D) spatial environments that represent real or non-real situations.” In a VR environment, learners can turn around and move in the same manner as they would in the real-world, and the digital environment responds to the learner’s actions in real time. In accordance with the different functional objectives on which VR systems focus, they can be divided into immersive and interactive systems (Vergara et al., 2017b; Johnson-Glenberg, 2018; Bagher et al., 2021; Makransky and Petersen, 2021; Huang et al., 2022; Petersen et al., 2022). Immersive VR provides a high level of objective sensory fidelity mainly through the use of head-mounted displays (Petersen et al., 2022). In the context of interactive virtual reality (IVR), users are usually allowed to explore, control, and even modify the virtual environment using hand-held controllers and virtual bodies, which offers them a high degree of freedom with respect to controlling the learning experience (Vergara et al., 2017b; Makransky and Petersen, 2021).

At present, the application of IVR in education remains in its early stages (Johnson-Glenberg, 2018), and there is a lack of research and application pertaining to the field of emotional value. However, due to increasing amounts of attention being given to experiential teaching, some of the affordability and sensory quality issues related to IVR have been addressed, and related technologies have become more mature and stable. The Gartner research report highlights the fact that the digital transformation and innovation in the context of education will be the main technological trends in higher education in 2022 (Morgan et al., 2022). The application of such technology to the learning environment and learning experiences will usher in new attention and opportunities for development (Morgan et al., 2022). Compared with immersive VR, the group collaboration, low costs, and convenience offered by IVR are more conducive to the mass adoption of this technology in education, such as in the case of units equipped with portable VR glasses (Vergara et al., 2017b). Moreover, evidence suggesting that IVR applications are beneficial for educational development is increasing (Mikropoulos and Natsis, 2011; Merchant et al., 2014; Araiza-Alba et al., 2022). Therefore, we have reason to believe that IVR experiential learning will become more common in educational settings. When demand arises, our researchers should be ready to provide effective theoretical and practical support.

IVR can support embodied learning because it offers a highly interactive environment that enables learners to gain embodied experience through the interactions among the body, its sensory motor system and the environment, thus allowing individuals to produce positive and relatively lasting changes in their behavior or behavioral potential. This process is known as “experiential learning involving feeling, perception, mind–body interaction and reaction” (Matthews, 1998). This interactive mode of multichannel perception, direct manipulation and real-time response represents an ideal state of embodied learning, which helps learners prolong memory time and promote knowledge transfer while improving their learning experience (Anastopoulou et al., 2011).

Although IVR could support embodied learning and relevant research has been conducted in related fields, for example, basic theoretical research (Mennecke et al., 2011), teaching experimental research (Tamaddon and Stiefs, 2017), and effect comparison research (Johnson-Glenberg et al., 2021), research on the internal mechanism of action associated with this process remains lacking. If IVR is to be used to support experiential learning, then it is necessary to study the relevant constructs and the relationships among them that can help achieve this goal. That is, we must answer the following question: “How does IVR allow learners to enhance their learning outcomes in the context of the learning experience?” The study of motivational theory (Renninger and Hidi, 2016; Wentzel and Miele, 2016) indicates that harnessing the emotional appeal of e-learning tools is significant for learning and instruction because the learner’s initial situational interest can be the first step in the promotion of learning (Renninger and Hidi, 2016), and the impact of VR features on learning outcomes is achieved indirectly *via* the learning experience (Lee et al., 2010; Makransky and Lilleholt, 2018).

To facilitate the effective use of IVR in education, this study considers relevant emotional experience factors that may affect learning outcomes, such as presence and perceived enjoyment. Presence represents learners’ psychological sense of “being there” in the environment constructed by the system (Lee et al., 2010; Makransky and Petersen, 2019). In VR environments, presence refers to an experiential feature of learners in the 3D virtual environment (Dalgarno and Lee, 2010). In educational virtual environments, presence can have a positive impact on learning outcomes (Lee et al., 2010; Mikropoulos and Natsis, 2011) and can play a key role in emotional experience (Makransky and Lilleholt, 2018). Perceived enjoyment refers to the degree to which learners believe that the VR environment is pleasant, interesting, and enjoyable (Tokel and Isler, 2015), and it is one of the important elements of emotional experience. According to the control value theory of achievement emotions (CVTAE, Pekrun, 2000; Makransky and Lilleholt, 2018), in the VR learning experience, the enjoyable emotions experienced by learners can have a positive impact on learning (Plass and Kaplan, 2016). Some studies have suggested that the impact of such positive emotions on learning achievement should be investigated, especially within interactive virtual learning environments (Dubovi and Lee, 2019; Skulmowski and Xu, 2021; Dubovi, 2022). To date, some studies have explored the impact of VR on learning outcomes by investigating psychological and emotional factors (Makransky and Lilleholt, 2018; Makki et al., 2019; Makransky and Petersen, 2021; Dubovi, 2022). However, research focusing on emotional experience in IVR remains scarce.

In summary, this study tries to identify the relevant constructs that play an important emotional role in an IVR-based learning environment that supports embodied learning as well as the relationships among these constructs. The study explores the ways in which IVR can enhance learning outcomes *via* emotional experiences. By understanding how these constructs work together to shape learning outcomes, we can develop targeted learning and visualization problems that are associated with appropriate burdens, and we can thus maximize the advantages of VR technology in this context (Salzman et al., 1999, p. 42; Lee et al., 2010). It can help IVR-based practitioners and educators understand the emotional value of this technology for improving learning outcomes.

## 2. Theoretical framework

The learning model based on immersive VR developed by Salzman et al. (1999) provides a starting point for the model constructs used in this study. Moreover, this model is supported by the media technology model developed of Lee et al. (2010). The technology media model proposed by Salzman et al. (1999) includes three main parts: input, process, and output. This model describes the importance of studying the ways in which VR features work in conjunction with other factors, including the concepts to be learned, the interactions that influence the learning process, and learning experiences, which in turn influence learning outcomes. The media technology model developed by Lee et al. (2010) explores the individual effects of psychological factors on learning in further detail and emphasizes the psychological learning process in the learning experience. This model provides a preliminary theoretical model for the determinants of learning outcomes in desktop VR-based learning environments.

The model constructs used in this study are also supported by the control value theory of achievement emotions (CVTAE, Pekrun, 2000; Makransky and Lilleholt, 2018). The CVTAE is a theoretical framework that describes the causal emotional process associated with the learning process in detail (Pekrun, 2000). The CVTAE believes that enjoyment, as a positive activity emotion, can promote learning (Pekrun, 2006; Pekrun and Stephens, 2010), which is an important emotional factor in the learning process. The research framework established by Makransky and Lilleholt (2018) is based on the CVTAE and the model developed by Lee et al. (2010). Makransky and Lilleholt distinguish between the affective variables and cognitive variables of the CVTAE, thus identifying two general paths that can describe the relationship between the individual’s degree of immersion and perceived learning outcomes in VR scientific simulation. This approach leads to a better understanding of the ways in which technical characteristics and students’ interactive experience affect important emotional and cognitive factors and the manner in which these relationships predict important educational outcomes.

**Figure 1:**
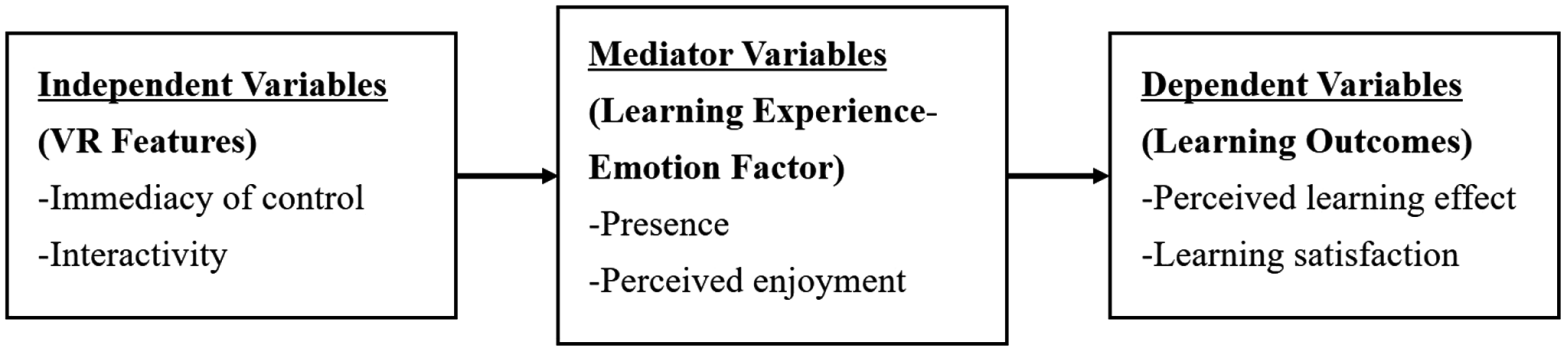
Conceptual framework of the outcomes and their causal relationships in an interactive virtual reality (IVR)-based learning environment.

Based on this theoretical framework, this study focuses on the use of interactive technology and emotional variables in the learning experience to understand the ways in which IVR technology promotes learning outcomes *via* emotional experience. Therefore, we construct a conceptual framework based on input, process, and output metaphors, emphasizing the psychology learning factors associated with the learning experience. This framework guides our research design and our evaluation of the way in which IVR can improve the learning effect, as shown in Figure 1, thus illustrating the conceptual framework of learning outcomes and their causal relationships in an IVR-based learning environment. In this framework, VR features affect learning outcomes indirectly *via* the psychological and emotional factors related to the learning experience. Thus, the input factor that may affect the learning process and the subsequent learning outcomes is IVR technology. IVR technology is evaluated based on its function. IVR features are used as independent variables, including the immediacy of control and interactivity. With regard to the process, we investigate the internal psychological emotional aspects of the learning experience, such as presence and perceived enjoyment, to identify the types of emotional learning experiences that are enhanced by IVR and to determine the importance of such emotional learning experiences to the learning outcomes. Hitherto, in the context of IVR-based learning, the influence of learners’ emotional perspectives on their learning outcomes has received little study. Finally, the learning outcomes based on IVR are measured in the context of emotions in terms of the perceived learning effect and learning satisfaction of students who engage in IVR learning. That is, this conceptual framework does not emphasize the direct impact of the VR function on learning outcomes but rather its indirect impact *via* emotional learning experience. The constructs and the correlations among their measurement variables are described below.

### 2.1. VR features

Studies have reported that VR features can affect learning outcomes (Lee et al., 2010; Makransky and Petersen, 2019; Petersen et al., 2022). In this study, we assume that the IVR function has an indirect influence on learning outcomes and that these influences are mediated by the learning experience. In other words, the quality of media (such as presence and perceived enjoyment) is considered the decisive factor in the learning experience. Regarding the distinctive functional features of VR, researchers have obtained some degree of developmental understanding, which provides a unique opportunity for the differential use of education. Hedberg and Alexander (1994) claim that immersion, fidelity, and active learner participation are the decisive features of VR. Lee et al. (2010) argue that VR features should be measured in terms of representational fidelity and immediacy of control, which have an impact on learning outcomes. The present view is that high-level immersion and interactivity are the decisive features of VR (Johnson-Glenberg, 2018; Makransky and Petersen, 2021; Petersen et al., 2022). According to a previous definition, immersion can be understood as the objective level of sensory fidelity provided by a VR system (Bowman and McMahan, 2007). The IVR learning environment used in this study, which is facilitated by portable VR glasses, cannot completely isolate users from the real-world and eliminate the influence of cues from the outside. It can be classified as exhibiting low immersion compared with head-mounted displays. Moreover, some studies have investigated the application of VR to engineering education. According to the results of a relevant survey, students demand using VR resources as frequently as possible to comply with the interactive design of teaching purposes to improve their learning experience (Vergara et al., 2016, 2017a,b). Furthermore, under conditions of low immersion, interactivity is more conducive to the learning experience and embodied learning (Petersen et al., 2022). Therefore, the IVR function on which this study focuses is measured in terms of the immediacy of control and interactivity. These factors constitute the unique features of an interactive VR learning environment and explain the educational value of IVR.

The immediacy of control refers to the ability of the user to change the position or direction of the view and the ability to pick up, interact with, and manipulate objects in the virtual environment. The immediate response to manipulation gives people the experience of moving smoothly in the virtual environment (Dalgarno et al., 2002; Lee et al., 2010). Interactivity indicates that users are given some degree of freedom (Makransky and Petersen, 2021) to control the learning experience. Intuitive interaction is an ideal feature of every educational environment, especially those that involve science (Araiza-Alba et al., 2022). In an interactive multimodal environment, learners can communicate well with the teaching environment system by using five common types of interaction, such as dialog, control, manipulation, search, and navigation (Moreno and Mayer, 2007). In summary, the decisive feature of interactivity is the ability to respond to the actions of learners during the learning process. In interactions that occur *via* dialog, learners can answer questions and obtain feedback. In interactions that occur *via* control, learners can determine the progress or order of the learning content. For example, learners can control the progress simply by using pause/play keys. Furthermore, the learner can zoom the view in or out, and the learner can move by using the front, back, left, and right buttons or by moving their own body when using wearable VR devices. In this study, we focus on dialog and control because they are the core features associated with the IVR environment. The study conducted by Witmer and Singer (1998) shows that the controlling factor is an important factor that affects interactive experience and learning and that a higher level of control leads to more active participation in the provision of interactive feedback (McMillan and Hwang, 2002). For example, real-time natural manipulations improve intuitive interaction (Araiza-Alba et al., 2022).

### 2.2. Affective factors of learning experiences

The affective factors of the learning experience used in this study include presence and perceived enjoyment. Witmer and Singer (1998) define presence as “the subjective experience of being in one place or environment, even when one is physically situated in another.” In other words, presence represents the subjective psychological response of learners to the system environment (Bowman and McMahan, 2007), and learners who have an experience of presence experience a psychological sense of “being there” in the environment constructed by the system (Lee et al., 2010; Makransky and Petersen, 2019). In VR environments, presence refers to an experiential feature of learners in the 3D virtual environment (Dalgarno and Lee, 2010). In a 3D environment, presence is produced by representational fidelity and a high degree of interactivity or user control rather than being merely a unique attribute of the environment (Dalgarno et al., 2002). Previous studies have shown that presence can have a positive impact on learning outcomes in educational virtual environments (Lee et al., 2010; Mikropoulos and Natsis, 2011) and can play a key role in emotional experience (Makransky and Lilleholt, 2018). Furthermore, the cognitive affective model of learning with media (Moreno and Mayer, 2007) suggests that an immersive VR environment could enhance presence by providing a more realistic experience (Slater and Wilbur, 1997). Petersen et al. (2022) found that both immersion and interactivity have positive effects on learners’ physical presence and embodied learning and that when immersion is low, interactivity has a greater mediating impact on the relationship between experience and embodied learning (Petersen et al., 2022). This is supported by the interest theories of learning starting with Dewey (1913), who argued that students learn through positive interactions with the environment. In other words, increased interactivity can lead to positive educational outcomes because the presence experienced by students can have a powerful emotional impact (Milk, 2015).

Perceived enjoyment has been defined as “the extent to which the learning activity is perceived to be enjoyable in its own right” (Davis et al., 1992). After development, in this study, perceived enjoyment refers to the degree to which learners believe that the VR environment is pleasant, interesting, and enjoyable (Tokel and Isler, 2015). According to the CVTAE, in the VR learning experience, the enjoyable emotions experienced by learners can have a positive impact on learning (Plass and Kaplan, 2016). In addition, perceived enjoyment is one component in fostering a sense of engagement and flow (Csikszentmihalyi, 2000), and it can provide a sense of perceived learning and satisfaction during a learning activity, which will lead to a positive intention to engage in similar activities (Makransky and Lilleholt, 2018).

### 2.3. Learning outcome variables

The learning outcome variables used in this study include learning satisfaction and perceived learning effect, while learning satisfaction refers to the degree to which students find the learning experience satisfactory (Makransky and Lilleholt, 2018). The perceived learning effect represents the degree to which the student perceives the learning experience as educational. These two outcome variables were included in the framework because this study explores the impact of technical characteristics and learning experience on learning outcomes in an IVR-based learning environment from the perspective of psychological emotional value. According to Sharda et al. (2004), the classification of learning outcomes can be divided into psychomotor outcomes, cognitive outcomes, and affective outcomes. Psychomotor outcomes include efficiency, accuracy, and response range. Cognitive outcomes include comprehension, knowledge, application, and analysis. Affective outcomes include learners’ perceptions of satisfaction with, attitudes regarding, and appreciation for the learning experience (Sharda et al., 2004; Lee et al., 2010). Therefore, the learning outcomes referenced by this study focus on the affective domain in terms of learning satisfaction and perceived learning effect with the IVR-based learning environment. In addition, previous studies identified these two factors as particularly relevant when using VR technology in an educational context (Lee et al., 2010).

## 3. Hypotheses

**Figure 2:**
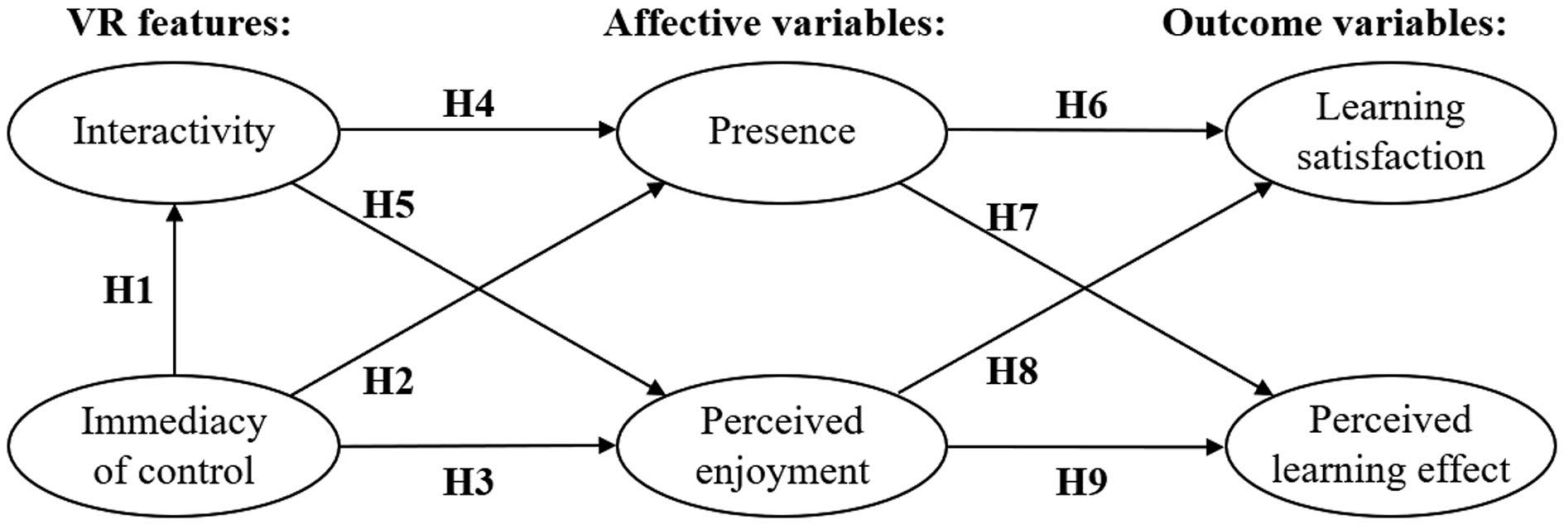
Hypothetical model.

Based on this conceptual framework, a hypothesized model for evaluating the ways in which IVR can improve learning outcomes is developed, as shown in Figure 2. Structural equation modeling (SEM) is used to evaluate the goodness-of-fit of the hypothesized model. The model focuses on the constructs and the causal relationships among them. The hypothesized model consists of (1) immediacy of control; (2) interactivity; (3) presence; (4) perceived enjoyment; (5) learning satisfaction; and (6) perceived learning effect. This study aims to investigate the ways in which the characteristics of IVR technology affect learning outcomes *via* psychological and emotional factors and to conduct more specific and in-depth research. Therefore, the immediacy of control and interactivity are not classified as a single VR feature construct, the presence and perceived enjoyment are not classified as a single learning experience construct, and learning satisfaction and perceived learning effect are not classified as a single learning achievement construct. Accordingly, the model can render an opaque construct (i.e., a construct in which certain specific influencing factors are considered jointly as a single construct) more transparent (i.e., by making the effect of each specific factor more visible), thus resulting in important implications and insights.

The relationship predicted in the previous model is given in Figure 2 and described in further detail below. Hypothesis-based theoretical models propose the following hypotheses to answer the research question: “How does IVR enhance learning outcomes *via* emotional experiences (perceived enjoyment and presence)?”

*H1*: Immediacy of control is significantly associated with interactivity.

*H2*: Immediacy of control is significantly associated with presence.

*H3*: Immediacy of control is significantly associated with perceived enjoyment.

*H4*: Interactivity is significantly associated with presence.

*H5*: Interactivity is significantly associated with perceived enjoyment.

*H6*: Presence is significantly associated with learning satisfaction.

*H7*: Presence is significantly associated with perceived learning effect.

*H8*: Perceived enjoyment is significantly associated with learning satisfaction.

*H9*: Perceived enjoyment is significantly associated with perceived learning effect.

## 4. Materials and methods

### 4.1. Context

With regard to the COVID-19 pandemic, the novel coronavirus exhibits high infectivity, rapid diffusion, a long incubation period, strong concealment, and a tendency toward easy variation; in addition, knowledge of pandemic prevention and control exhibits the characteristics of abstractness, complexity, and negligence. As a result, most people’s knowledge in this context lacks a scientific and systematic basis, and so they cannot cooperate scientifically with pandemic prevention and control measures, especially in densely populated places, such as schools. Therefore, during the spring semester of 2022, China West Normal University offered a popular science course on pandemic prevention and control knowledge that included an IVR-based COVID-19 pandemic science museum. Simultaneously, an empirical survey was conducted to investigate the associated learning outcomes and processes. This approach can greatly enhance the scientificity and effectiveness of pandemic prevention and control.

### 4.2. Participants and procedure

The sample referenced by this study comprised 480 typical college students from a Chinese university, including 193 males and 287 females between the ages of 18 and 25 years. The students learned about pandemic prevention and control by visiting the IVR-based COVID-19 pandemic science museum. Each session of the learning activity was attended by 10 learners and one teacher simultaneously, and students in each group were required to wear VR glasses. Students in each group were shown the glasses and guided by a teacher wearing the main-view VR glasses. Each learning activity lasted approximately half an hour. Throughout the learning experience, first, the teachers wore the main-view glasses to demonstrate and guided the operation of the VR controller and the main-view glasses, as well as how to visit; then, the students chose to wear the main-view glasses in turn for experiential learning according to their individual learning needs; at the same time, group members could communicate the learning content and discuss their progress with each other. Subsequently, each member participated in the following questionnaire survey, which focused on input (VR function), process (learning experience), and output (learning outcomes). That is, the variables included immediacy of control and interactivity, presence and perceived enjoyment, and learning satisfaction and perceived learning effect. In the experiment, the students were informed of the nature of the study and participated voluntarily.

### 4.3. Materials

#### 4.3.1. Measurements

The questionnaire included independent variable associated with VR function (immediacy of control and interactivity); mediating variables pertaining to learning experience (presence and perceived enjoyment); and dependent variables focused on learning outcomes (learning satisfaction and perceived learning effect). The complete list of items and the sources of the scales used are included in Appendix A.

Immediacy of control was measured using 4 items adapted from Dalgarno et al. (2002) and had a Cronbach’s alpha coefficient of 0.849. An example item was “being able to manipulate objects in real time in a virtual environment is better for my understanding.”

Interactivity was measured using 4 items adapted from McMillan and Hwang (2002) and had a Cronbach’s alpha of 0.789. An example was “I can easily manage my learning progress in this virtual reality/computer-based learning environment.”

Presence was measured using 4 items adapted from Makransky and Lilleholt (2018) and had a Cronbach’s alpha of 0.910. An example item was “the feeling of my movement in a virtual environment is very real.”

Perceived enjoyment was measured using 3 items adapted from Tokel and Isler (2015) and had a Cronbach’s alpha of 0.930. An example item was “I enjoy using virtual reality to learn.”

Learning satisfaction was measured using 5 items adapted from Lee et al. (2010) and had a Cronbach’s alpha of 0.862. An example item was “I am satisfied with the overall learning effect.”

Perceived learning effects were measured using 4 items adapted from Lee et al. (2010) and had a Cronbach’s alpha of 0.867. An example item was “I learned to identify important and major problems regarding this topic.”

All items were rated on a 5-point Likert scale ranging from strongly disagree to strongly agree.

#### 4.3.2. IVR-based pandemic science museum

**Figure 3:**
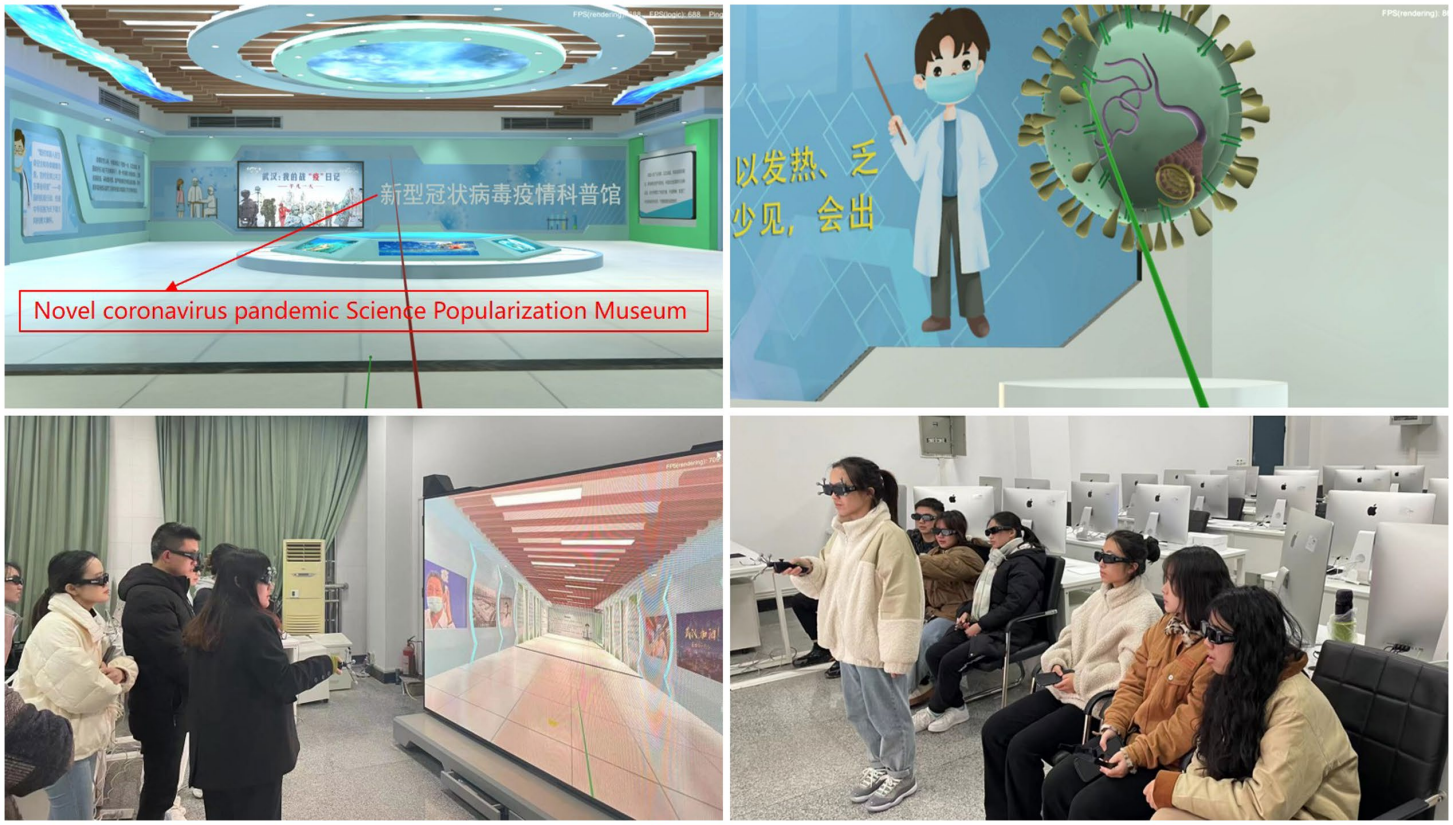
The IVR pandemic science museum simulation scenes and activities carried out map.

The IVR-based COVID-19 pandemic science museum was customized by China West Normal University for Shanghai ManHeng Digital Technology Co., Ltd., and used as learning material. The scenes and activities associated with this museum are shown in Figure 3. By facilitating experiential learning, the museum helps learners master information regarding the prevention and control of the COVID-19 pandemic more systematically and efficiently. Furthermore, this approach helps improve learners’ awareness of and capabilities with respect to self-protection, thereby reducing the risk of virus infection and preventing panic.

The IVR system consists of IQ-Tracker tracking, an LED display, a graphics workstation, a sound system, 11 VR glasses (1 main-view) and a VR controller. Both the main-view VR glasses and the VR controller have a signal transmitting ball, which can be used to locate their own position with the tracker. When the VR controller points to the LED display, the program generates an indicator line on the screen that the operator can use to point to a text, a picture, or a detail of a model in the museum. In the COVID-19 pandemic science museum, 2D video windows are embedded in 3D scenes, and while each section of the pandemic prevention and popular science documentary is played, learners can control the playback progress. Simultaneously, the simulation features an exhibition of COVID-19 pandemic knowledge using graphics, images and text, and an interactive intelligent robot is included in the simulation to broadcast and explain this information. Learners can touch and manipulate learning objects directly or indirectly using bodily movements or third-party media. For example, learners wearing main viewing glasses can change the size and position of the VR viewing angle by rotating their head or moving their body; learners can achieve the five operational requirements of movement, steering, pointing, selection and menu navigation through the VR controller. Learners communicate with the learning environment system mainly by dialog and control. For example, learners can point to learning content and get intelligent broadcasts or answer preset window questions and receive timely feedback; learners can determine the progress and order of learning plots through control. That is, multichannel perceptions, direct control, and real-time responses facilitate interaction, thus allowing learners to complete the relevant learning experience in the VR science museum.

The simulation experience begins with the display of a 2D documentary regarding the pandemic at the Science Center. Subsequently, learners advance interactively through the simulation and visit a circular science popularization corridor. Relevant pandemic prevention and control knowledge and 3D objects are displayed in the air and on the walls on both sides of the corridor. Finally, learners choose whether to initiate the answer mode, according to which a simple test including 10 questions measures users’ mastery of basic knowledge regarding COVID-19 prevention and control. Throughout the whole process of the simulation experience, self-pacing and guided learning principles are adopted to enhance perceived learning outcomes.

### 4.4. Data collection and analysis

It takes about 15 min for each subject to fill out the questionnaire. The data referenced by this study were collected in January 2022. First, the questionnaire was uploaded to WJX.^1^ Subsequently, we performed data entry and management using Excel 2010 software and conducted descriptive statistical analysis and correlation analysis using SPSS 21.0 software. Finally, SEM analysis was conducted using Mplus 8.3 software, and the relationships in the model were tested.

## 5. Results

### 5.1. Descriptive statistics and validity and reliability analysis

In this study, the means, standard deviations, CR (Composite Reliability), AVE (Average Variance Extracted), and Pearson correlation coefficients of VR features (i.e., immediacy of control and interactivity), the learning experience emotion factor (i.e., presence and perceived enjoyment), and learning outcomes (i.e., perceived learning effect and learning satisfaction) were analyzed (Table 1).

**Table 1:** Descriptive statistics and correlation analysis for each variable.

Variable	M	SD	CR	AVE	1	2	3	4	5	6
1. Immediacy of control	4.32	0.52	0.75	0.43	–					
2. Interactivity	4.25	0.66	0.86	0.61	0.69**	–				
3. Presence	4.12	0.65	0.81	0.53	0.60**	0.60**	–			
4. Perceived enjoyment	4.37	0.58	0.74	0.49	0.55**	0.43**	0.51**	–		
5. Learning satisfaction	4.29	0.55	0.82	0.47	0.62**	0.56**	0.65**	0.67**	–	
6. Perceived learning effect	4.14	0.64	0.83	0.54	0.60**	0.64**	0.72**	0.51**	0.71*	–

M, mean; SD, standard deviation; *p < 0.05, **p < 0.01.

The average variance extracted (AVE) is higher than 0.5, but we can accept 0.4. Because Fornell and Larcker said that if AVE is less than 0.5 but composite reliability is higher than 0.6, the convergent validity of the construct is still adequate (Fornell and Larcker, 1981; Lam, 2012). All six factors involved in this study have AVE values greater than 0.4 and CR values greater than 0.7, which shows that the scale data measured this time has excellent aggregation. In addition, the results showed that perceived learning effect was positively linked with VR features (*r* = 0.60, *p* < 0.01; *r* = 0.64, *p* < 0.01) and the learning experience emotion factor (*r* = 0.72, *p* < 0.01; *r* = 0.51, *p* < 0.01). Learning satisfaction was positively linked with VR features (*r* = 0.62, *p* < 0.01; *r* = 0.56, *p* < 0.01) and the learning experience emotion factor (*r* = 0.65, *p* < 0.01; *r* = 0.67, *p* < 0.01). Perceived enjoyment was positively linked with VR features (*r* = 0.55, *p* < 0.01; *r* = 0.43, *p* < 0.01) and presence (*r* = 0.51, *p* < 0.01). Presence was positively linked with VR features (*r* = 0.60, *p* < 0.01; *r* = 0.60, *p* < 0.01). Immediacy of control was positively linked with interactive feedback (*r* = 0.69, *p* < 0.01).

### 5.2. Structural model results

**Figure 4:**
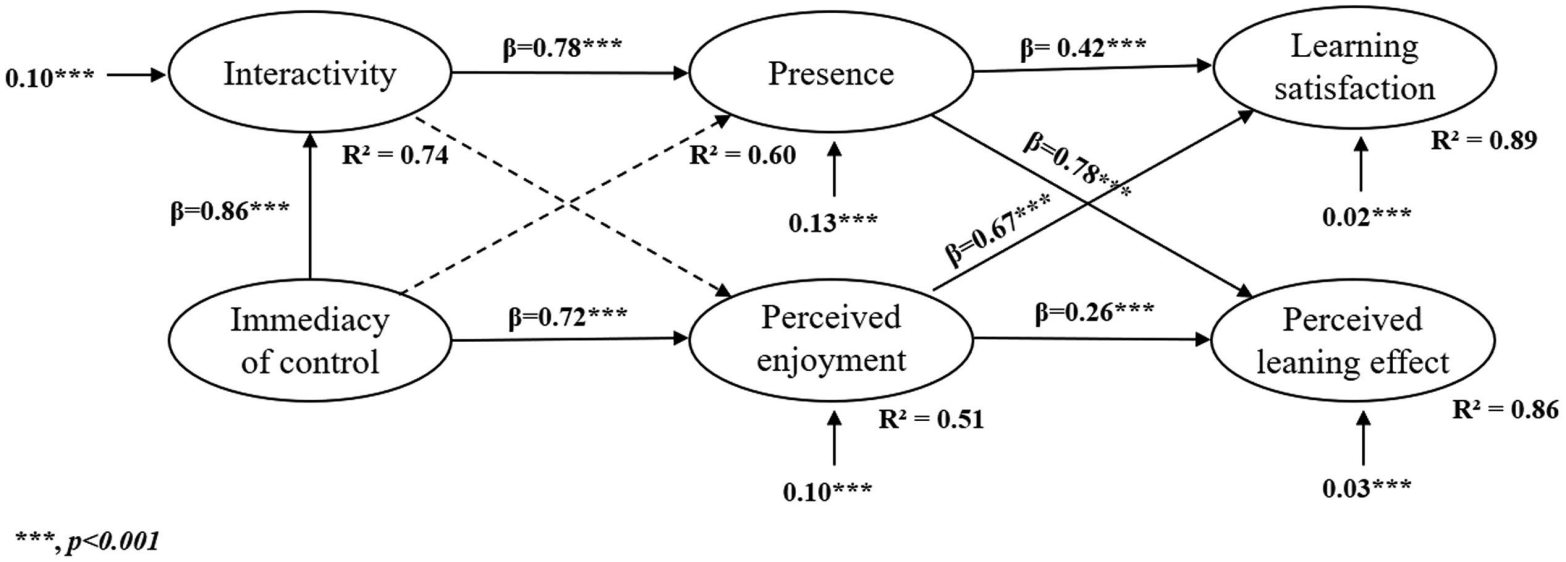
Structural model with results.

This study takes the VR characteristics as independent variables, emotional variables as intermediary variables, and result variables as dependent variables to construct a mediating model. This model uses the χ^2^ value by the degrees of freedom (χ^2^/df), standardized residual mean root (SRMR), and root mean square error of approximation (RMSEA), comparative fit index (CFI), and Tucker–Lewis index (TLI) to evaluate the fit index for this study. Table 2 lists the recommended and actual values of the model-fitting index for our model. All the fitting indices meet the appropriate standard, thus proving that our model fits the data well. The path coefficients of the structural model are shown in Figure 4.

**Table 2:** Fit indices and recommended values of structural model.

Fit index	χ^2^/df	RMSEA	CFI	TLI	SRMR
Recommended range	<3	<0.08	>0.9	>0.9	<0.08
Measured value	2.55	0.06	0.93	0.92	0.05

RMSEA, root mean square error of approximation; CFI, comparative fit index; TLI, Tucker–Lewis index; SRMR, standardized residual mean root.

In social science studies, common method variance is normal due to the data collection procedures. We run the Harman one-factor test to evaluate the effect of common method variance on the constructs of the study (Podsakoff et al., 2003). The result of one-factor Harman’s test revealed that common method variance is not a critical matter in this study because the main factor explained 42.80% of the variance, indicating less than the suggested limit of 50% (Fuller et al., 2016).

### 5.3. Analysis of the hypotheses

A confirmatory factor analysis was conducted to test the fit of the hypothesized relationships among the constructs in the *a priori* model shown in Figure 2. This hypothesized model nearly attained an acceptable fit (RMSEA = 0.05, CFI = 0.94, TLI = 0.93) but contained several nonsignificant paths, which were deleted by an iterative procedure. Each of these paths was evaluated and removed successively based on the greatest degree of misfit until all remaining paths were significant. This process resulted in a simplified model that contained only significant loadings (see Figure 4). The final simplified model shown in Figure 4 obtained an acceptable fit (RMSEA = 0.06, CFI = 0.93, TLI = 0.92). Furthermore, all the standardized path coefficients shown in Figure 4 are significant at the alpha level of 0.001. Below, we present the results concerning each of the 9 hypotheses proposed in this study.

#### 5.3.1. H1: Immediacy of control is significantly associated with interactivity

H1 was supported. As expected, immediacy of control is a significant antecedent of immediacy (*β* = 0.86, *p* < 0.001).

#### 5.3.2. H2: Immediacy of control is significantly associated with presence

H2 was not supported. Immediacy of control is not significantly associated with presence, thus indicating that the immediacy of control of VR had little effect on the presence of the experimenter in our study.

#### 5.3.3. H3: Immediacy of control is significantly associated with perceived enjoyment

H3 was supported. Based on the available data, immediacy of control is positively correlated with perceived enjoyment (β = 0.72, p < 0.001).

#### 5.3.4. H4: Interactivity is significantly associated with presence

H4 was supported. As expected, according to this study, a change in interactivity is significantly related to a change in presence (*β* = 0.78, *p* < 0.001).

#### 5.3.5. H5: Interactivity is significantly associated with perceived enjoyment

H5 was not supported. Interactivity is not significantly associated with perceived enjoyment.

#### 5.3.6. H6: Presence is significantly associated with learning satisfaction

H6 was supported. As expected, presence is positively correlated with learning satisfaction (*β* = 0.42, *p* < 0.001).

#### 5.3.7. H7: Presence is significantly associated with perceived learning effect

H7 was supported. The results of this study indicate that presence is positively correlated with perceived learning effect (*β* = 0.78, *p* < 0.001).

#### 5.3.8. H8: Perceived enjoyment is significantly associated with learning satisfaction

H8 was supported. Perceived enjoyment is positively correlated with learning satisfaction (*β* = 0.67, *p* < 0.001).

#### 5.3.9. H9: Perceived enjoyment is significantly associated with perceived learning effect

H9 was supported. The results indicate that perceived enjoyment is positively correlated with perceived learning effect (*β* = 0.26, *p* < 0.001).

### 5.4. Mediating effect of the research model

Table 3 displays the results of the mediation tests. Specifically, we used the bootstrap method to test the mediating effect and obtained 5,000 samples with a 95% confidence interval (CI). The bootstrap method does not assume that the mediating effect is normally distributed, and it is suitable for application to small or medium-sized samples (Cheng et al., 2020). The results of the bootstrapping are shown in Table 3. When the upper and lower limits of the CI do not contain zero, the path contains a mediating effect. First, presence had mediating effects on the relationship between interactivity and learning satisfaction as well as on the relationship between interactivity and perceived learning effect. Second, perceived enjoyment had mediating effects on the relationship between immediacy of control and learning satisfaction as well as on the relationship between immediacy of control and perceived learning effect. These results suggest that emotional experiences (perceived enjoyment and presence) play a key mediating role in the relationship between VR technology features and perceived learning outcomes.

**Table 3:** Standardized bootstrap mediation test.

	Point estimate	Product of coefficients			BOOTSTRAP 5000 TIMES 95% CI			
					Bias corrected		Percentile	
Path		S.E.	Est./S.E.	*P*-value	Lower	Upper	Lower	Upper
IOC → PE → LS	0.55	0.09	6.42	0.000	0.40	0.75	0.40	0.74
IOC → PE → PLE	0.21	0.06	3.45	0.001	0.11	0.36	0.10	0.35
INT → PRE → LS	0.53	0.10	5.29	0.000	0.37	0.78	0.37	0.77
INT → PRE → PLE	0.49	0.07	7.55	0.000	0.37	0.62	0.37	0.62

IOC, immediacy of control; INT, interactivity; PR, presence; PE, perceived enjoyment; LS, learning satisfaction; PLE, perceived learning effect.

## 6. Discussion

### 6.1. Empirical contributions

According to the results of the SEM, the use of the IVR function in popular science-based learning related to COVID-19 indirectly predicts the perceived learning effect *via* emotional experience, as expected based on our previously developed model; however, not all prior predictions are significant. The results of this study show that in a virtual learning simulation, emotional elements entailed by the learning experience, such as presence and perceived enjoyment, have a key mediating effect on the relationship between IVR features and perceived learning effect. This finding is the major empirical contribution of this study, which provides a theoretical framework for studying the results of IVR learning in the context of emotions.

#### 6.1.1. The role of presence in emotional experience

The data results support our hypothesis that the characteristics of IVR technology, that is, the interactivity and immediacy of control provided by the environment, are related to the availability of greater presence in the IVR-based learning experience. In turn, the improvement in presence is related to an increase in the learning effect and learning satisfaction; that is, a positive change in presence is related to improved learning outcomes.

These findings suggest that presence plays a leading role in the learning experience (Winn et al., 2002; Mikropoulos and Natsis, 2011). The results of this study suggest that the presence associated with the VR learning environment can affect the learning process, which is consistent with the findings of other researchers (Witmer and Singer, 1998; Dubovi et al., 2017; Dubovi, 2022), and can allow participants to interact with the VR environment in a more natural way, thus leading to better learning outcomes (Lee et al., 2010; Araiza-Alba et al., 2022). Presence positively predicts learning outcomes such as learning satisfaction and perceptual learning. This research conclusion is consistent with the findings of Makransky and Lilleholt (2018) and Lee et al. (2010), who view learning satisfaction and perceived learning effect as the latent variables of learning outcomes (the dependent variable) within the evaluation, and the results indicate that presence has a positive predictive effect on learning outcomes. In this study, we take learning satisfaction and perceived learning effect as dependent variables directly to represent learning outcomes in the context of further research. The research data show that the correlation between presence and perceived learning effect is significantly greater than the correlation with learning satisfaction. This finding is not merely a further verification of previous results and a supplement to previous studies; it also clarifies the relationships among specific elements, which is convenient for targeted intervention practice. Simultaneously, this finding represents an important empirical contribution of this study. Buttussi and Chittaro (2017) and Makransky et al. (2019) indicating that presence in VR learning is unrelated or negatively related to learning effects, which is inconsistent with our findings. However, some researchers, by reference to EEG measurement data, have explained that immersive VR not only produces a high sense of presence but also leads to a high external cognitive load (Makransky et al., 2019; Baceviciute et al., 2021). The IVR referenced by this study offers a variety of functions that facilitate either direct interaction or indirect interaction, which can enable learners to familiarize themselves with and integrate themselves into the environment quickly, which can help reduce irrelevant cognitive load (Albus et al., 2021).

After data verification, the level of presence experienced by learners can be affected directly by the interactivity of VR and indirectly by the immediacy of control *via* interactivity. IVR offers an interactive mode of multichannel perception, direct manipulation and real-time response by providing direct and indirect interaction functions, which allows learners to explore and manipulate the virtual environment freely. In this study, learners are guided to interact with the physical content or manipulate the content in the scene, which requires them to activate more sensorimotor areas, thus allowing for enactive engagement and high levels of embodiment in the learning experience (Johnson-Glenberg, 2019). The interactive characteristics of the 3D virtual environment are an important antecedent of the characteristics of the interactive experience of learners, which can enhance their learning experience and provide them with more opportunities to engage in experiential learning (Dalgarno and Lee, 2010). Simultaneously, these interactive characteristics can also help learners prolong memory time and promote the transfer of knowledge (Anastopoulou et al., 2011). The results reported by Ferguson et al. (2020) based on controlled experiments in VR games also show that allowing players to navigate freely in the game (one type of interaction) has a positive effect on presence. A recent survey of immersive VR-based and desktop VR-based learning processes conducted by Petersen et al. (2022) also shows that interactivity has a positive impact on the presence experienced by learners. The conclusion highlights the fact that both immersion and interactivity have positive effects on learners’ physical presence and embodied learning, but the interaction effect indicates that when immersion is low, interactivity has a greater mediating impact on the relationship between experience and embodied learning (Petersen et al., 2022). Accordingly, in this case, users tend to feel immersed and not to pay attention to real-world cues when engaging in efficient interactive experiences in IVR-based environments featuring low immersion, a situation which is more conducive to learning experiences and embodied learning. In this study, we further validate this conclusion in an IVR-based environment, which is also one of the empirical contributions of this research. The result indicating that the immediacy of control affects presence indirectly *via* interactivity can be explained by reference to Witmer and Singer (1998), who claim that the control factor (i.e., the amount of control that the user has in the VR environment) is an important factor that affects the interactive experience and learning. Building on this foundation, studies conducted by Lee et al. (2010) and Makransky and Lilleholt (2018) manipulate the control factor directly in terms of the immediacy of control.

However, this study shows that presence cannot be affected by the immediacy of control of VR directly. This conclusion is different from the findings reported by Makransky and Petersen (2019) and Makransky and Petersen (2021). Based on desktop VR and immersive VR respectively, these studies claim that the immediacy of control can predict presence directly. This finding can be explained by reference to the model of learning in 3D virtual environments developed by Dalgarno et al. (2002) and Dalgarno and Lee (2010); that is, presence is not a unique attribute of 3D virtual environments but rather a characteristic of learners’ experience in the context of these environmental characteristics. Slater (2009) and Slater et al. (2009) note that for a VR system to generate a higher sense of presence, it must provide nearly real-time interactive feedback between the user’s behavior and sensory systems. Accordingly, it can be concluded that interactivity, as a direct antecedent of presence, has a mediating effect on the relationship between the immediacy of control and presence. In other words, good immediacy of control does not necessarily entail a high sense of presence; rather, only when good interactivity is triggered by good immediacy of control can the learner’s presence be affected by a good interactive experience. This finding also represents an empirical contribution of this study.

#### 6.1.2. The role of perceived enjoyment in emotional experience

The results support the hypothesis that the immediacy of control associated with IVR is related to learners’ perceived enjoyment and that in turn, an improvement in enjoyment is related to an increase in the learning effect and learning satisfaction; that is, a positive change in enjoyment is related to improved learning outcomes.

Such findings suggest that enjoyment plays a key role in influencing learning outcomes. In the IVR-based environment referenced by this study, learners can be guided to explore freely. This result is consistent with previous findings that VR-based environments can trigger more positive emotional expressions, such as enjoyment (Dubovi and Lee, 2019). Emotional expressions may indicate higher levels of engagement with the learning content, which in turn correlates with learning outcomes (Pekrun and Linnenbrink-Garcia, 2012). Studies have shown that harnessing the emotional attractiveness of e-learning tools is a central issue for effective instruction and learning, as initial situational interest can be the first step in facilitating learning (Renninger and Hidi, 2016; Wentzel and Miele, 2016). In this study, the IVR-based COVID-19 pandemic science museum offers an engaging, novel, fun, and relaxed environment by means of 3D visualization, intelligent voice broadcast and explanation, 2D video, background music, flat images, and so on. This environment can stimulate learners’ curiosity, imagination, and inspiration, broaden their horizons, and thus enhance their perceived enjoyment. In addition, the conclusions drawn by this study have also been proven and explained by similar studies; for example, in immersive VR, learners’ perceived enjoyment can predict learning outcomes such as behavioral intention, perceived learning, and learning satisfaction, which is consistent with the result of Makransky and Lilleholt (2018). In computer-supported collaborative learning (CSCL) environments, students’ perceived enjoyment has a significant positive impact on their learning satisfaction and a perceived impact on their learning (Muñoz-Carril et al., 2021). Serrano-Cámara et al. (2014) also confirm that students who experience enjoyment in the online environment are more satisfied with the learning process and believe that it has an effective impact on their academic development. In addition, the data referenced by this study indicate that the correlation between perceived enjoyment and learning satisfaction is significantly greater than the correlation with perceived learning effect. This finding is also a meaningful empirical contribution of this study that can facilitate the development of targeted intervention practices. Overall, these studies have validated the claim of the CVTAE that learning can be facilitated by positive activity emotions such as enjoyment (Pekrun, 2006; Pekrun and Stephens, 2010).

The premise underlying the associations among enjoyment, positive emotions and learning is that students have a sense that they are able to master the material (Dubovi and Lee, 2019). According to the CVTAE, the extent to which students experience a sense of control regarding educational content can produce enjoyment (Plass and Kaplan, 2016). Research conducted by Roseman and Smith (2001) and Caglar-Ozhan et al. (2022) also indicates that the evaluation of control potential is a component that affects emotion directly. Thus, enjoyment can be predicted to be stronger when the learning activity in question is sufficiently controllable (Pekrun, 2006). In other words, the immediacy of control is positively correlated with the learner’s perceived enjoyment. In this study, the IVR system is supported by high-precision IQ-Tracker tracking technology, a high-definition LED display screen, and a high-response graphics workstation, which ensure the immediacy of control. Other studies have come to the same conclusion by investigating immersive VR-based and desktop VR-based learning processes (e.g., Makransky and Lilleholt, 2018; Makransky and Petersen, 2019). Simultaneously, the studies conducted by Lin and Lin (2019) and Muñoz-Carril et al. (2020) also confirm this conclusion indirectly. These studies conclude that positive attitudes toward the use of technology-based learning methods, such as CSCL, can increase students’ perceived enjoyment in the context of learning by using a variety of tools. However, these findings fail to support the hypothesis that the interactive features of VR predict perceived enjoyment. Based on this study, we believe that the reason underlying this conclusion may be that the learner experiences a more pronounced sense of direct control but a weaker internal sense of connection to the interactivity that is affected by the immediacy of control.

### 6.2. Practical implications

Based on the theoretical framework offered by the CVTAE (Pekrun, 2000; Makransky and Lilleholt, 2018) and technology-mediated learning (Salzman et al., 1999; Lee et al., 2010), this study provides a model for understanding the affective factors that affect interactive VR-based simulation learning as well as the relationships among those factors. We highlight the way in which technological features of IVR may impact learners’ presence and perceived enjoyment and, in turn, how these emotional experiences may affect learners’ perceived learning outcomes.

This study has three practical implications. It can serve as a reference for practitioners and educators who wish to use IVR to facilitate experiential teaching, constructing virtual simulation experiment platforms, and driving the digital transformation and innovation of educational instruction. First, the research results indicate that when designing an IVR-based environment, the immediacy of control and interactivity are two important variables that require attention. Specifically, the degree of freedom given to learners to allow them to control the learning experience is directly related only to presence, while the ability to control various factors in the virtual environment is directly related to the learners’ perceived enjoyment and indirectly related to presence *via* interactivity. Therefore, we can ensure high-quality IVR functions in two ways during the design process. The first way is to improve interactivity by presenting the material in a more logical manner, designing engaging sessions, providing kinder learning feedback, and including more diverse types of interaction, such as dialog, search, and navigation (Moreno and Mayer, 2007). Another way is to improve the immediacy of control by allowing for real-time object manipulation, a smooth display of view position and changes in the motion of objects, 3D audio and imaging technology as well as a high degree of view control (Dalgarno and Lee, 2010; Makransky and Petersen, 2019).

Second, the findings suggest that when designing VR-based learning activities, the emotional experience of learners (i.e., perceived enjoyment and presence) should be emphasized. Specifically, the degree to which learners have a psychological sense of “being there” in the VR environment is related to their perceived learning outcomes, and the degree to which learners find the VR environment to be pleasant, fun, and enjoyable is also related to their perceived learning outcomes. In other words, learners with high levels of presence and enjoyment exhibit greater improvement in terms of learning satisfaction and perceived learning effect. Therefore, we can ensure a high level of emotional experience in two ways in activity design. The first way is to facilitate presence by offering scenarios that are more closely related to real life and emphasizing the scientific, artistic, educational, and technical principles of VR activities. Another way is to enhance perceived enjoyment. For example, according to the primacy effect, a novel and unique theme should be established to ensure that learners receive a deep first impression. Likewise, based on the tailing effect, learners’ enjoyment and memory can be improved by summarizing and reflecting at the end of the activity, such as taking pictures. In addition, in the context of learning activities, a reasonable reward mechanism can be implemented to drive game-based teaching.

Third, the data results show that when designing learning outcome interventions, presence and enjoyment are two relevant emotional variables. Specifically, in the learning experience, the correlation between the level of presence and the learning effect is significantly greater than the correlation with learning satisfaction, while the correlation between the level of enjoyment and learning satisfaction is significantly greater than the correlation with the learning effect. In other words, we can control learners’ perceived learning effect more effectively *via* presence, and we can control learning satisfaction more effectively *via* learners’ perceived enjoyment. Therefore, when developing targeted interventions, our practitioners can design teaching plans or experiments based on different significant paths. This approach can help us develop a data-driven comprehensive evaluation of education more effectively.

## 7. Limitations and directions for future research

This study faced three limitations. First, the effect of individual differences in terms of the relevant variables on learners’ perceived learning outcomes was not assessed. Certain individual variable-related differences can moderate the impact of the technical learning interventions discussed in this study, including vertigo, spatial ability, cognitive style, learning motivation, self-efficacy, and learning attitude (Makransky and Petersen, 2021; Hwang and Chien, 2022). To ensure the depth and controllability of this study and to reduce the length of the questionnaire, these individual factors were not taken into account. Second, the influence of cognitive load on emotional experience was not considered. In IVR-based learning environments, information capacity is greatly improved due to the associated increase in visual range and interactivity, which may lead to distraction, increase unnecessary cognitive load, and reduce the effectiveness of the learning experience (Parong and Mayer, 2021). Third, the sample range and learning topics used in this study faced certain limitations. In this study, college students were selected to engage in experiential learning in an IVR-based COVID-19 pandemic science museum. Therefore, the sample size was insufficiently large and wide. Moreover, the COVID-19 pandemic science museum mainly involves actual knowledge, conceptual knowledge, and procedural knowledge but does not address migratory knowledge.

Based on the current research findings and the remaining limitations, we propose the following research directions that can be pursued in the future:

1. The impact of individual differences in terms of variables on learning experiences in an IVR-based environment represent an important direction for future research.
2. The impact of social cues, such as textual cues or visual cues, on the interactive VR-based learning process is an important direction for future research (Albus et al., 2021; Liu et al., 2021). According to the signal principle, adding clues to multimedia learning materials can highlight key contents, guide learners’ attention, and reduce cognitive load (Gog, 2014; Karich et al., 2014).
3. Future research should investigate whether the model framework constructed in this study can be generalized to other learner samples and different learning topic scenarios.

## 8. Conclusion

In this study, we explored the internal mechanism by which IVR features affect learning outcomes *via* learning experience based on media technology models and the CVTAE. Unlike previous studies, we focused on IVR environments and emphasized the interactive effect of VR features and learning experience on learning outcomes from the perspective of psychological and emotional value. By using SEM, the key mediating effect of emotional experience (presence and perceived enjoyment) on the relationship between IVR features and perceived learning outcomes was explored, which facilitated the development of a preliminary theoretical model of the emotional factors that influence learning outcomes in an IVR learning environment. In addition, by reference to the data thus analyzed, we highlighted the different effects of presence and enjoyment on learning satisfaction and perceived learning effect in the IVR learning experience.

Practitioners and educators can benefit from this study because, first, these results can provide intellectual and theoretical support for VR teaching design and VR environment development for educational purposes, such as by helping to develop a training platform for educational virtual simulation experiments. Second, this study expands the applicability of embodied learning theory to different scenarios, and the research results thus have practical value for the large-scale application of IVR in the future with the aim of providing experiential and group-led teaching and promoting the digital transformation and intelligence upgrading in education. Third, this study proposes a preliminary theoretical model of the emotional factors that influence learning outcomes in the context of IVR learning experience, which can help scholars in this field conduct further related research on this basis.

## Data availability statement

The original contributions presented in the study are included in the article/Supplementary material, further inquiries can be directed to the corresponding author.

## Ethics statement

The studies involving human participants were reviewed and approved by the Ethics Committee of China West Normal University. The patients/participants provided their written informed consent to participate in this study. Written informed consent was obtained from the individual(s) for the publication of any potentially identifiable images or data included in this article.

## Author contributions

HY and YD: research design, methodology, and funding acquisition. RL and LL: material preparation, data collection, and reference management. HY and MC: data analysis and critical manuscript revisions. QX: writing – review, editing, and manuscript revision. All authors contributed to the article and approved the final draft submitted.

## Funding

This study was supported by the General Project of Teaching Reform of Sichuan Education Department (JG2021-947), the Doctoral Project of China West Normal University (21E007), the Projects of Industry-University Collaborative Education of the Ministry of Education (202102464026), the Project of High-quality Development of Sichuan compulsory Education (YWZDWT-2022-02), and the Project of Nanchong Social Science Planning (NC2020B227).

## Conflict of interest

The authors declare that the research was conducted in the absence of any commercial or financial relationships that could be construed as a potential conflict of interest.

## Publisher’s note

All claims expressed in this article are solely those of the authors and do not necessarily represent those of their affiliated organizations, or those of the publisher, the editors and the reviewers. Any product that may be evaluated in this article, or claim that may be made by its manufacturer, is not guaranteed or endorsed by the publisher.

## Supplementary material

The Supplementary material for this article can be found online at: https://www.frontiersin.org/articles/10.3389/fpsyg.2022.1081372/full#supplementary-material

## Footnotes

^1^ www.wjx.cn/
