# Supplementary material for: How does interactive virtual reality enhance learning outcomes via emotional experiences? A structural equation modeling approach
Source: Front Psychol. 2023 Jan 6;13:1081372. doi: 10.3389/fpsyg.2022.1081372 (PMC9853441; doi:10.3389/fpsyg.2022.1081372)
Supplement: Supplementary file 1 [file Table_1.DOCX]

Supplementary Material

1. **Appendix A**

| Questionnaire items and sources | | |
| --- | --- | --- |
| **Measurements** | **Items** | **Sources** |
| Immediacy of control | 1. Being able to change the view position of a 3D object helped me learn better. | Dalgarno et al. (2002) |
|  | 2. The ability to change the view position of a 3D object makes learning more dynamic and fun. |  |
|  | 3. Being able to manipulate objects in a virtual environment makes learning more motivating and fun. |  |
|  | 4. Being able to manipulate objects in real time in a virtual environment is better for my understanding. |  |
| Interactivity | 1. The contents, forms and situations in this VR learning environment are rich and varied. | McMillan and Hwang (2002) |
|  | 2. I can easily manage my learning progress in this virtual reality/computer-based learning environment. |  |
|  | 3. I can easily pay attention to the items of knowledge in which I am interested in this VR environment. |  |
|  | 4. I can receive real-time feedback in this virtual reality/computer-based learning environment. |  |
| Presence | 1. I can interact with the virtual environment naturally. | Makransky and Lilleholt (2018) |
|  | 2. My experience in the virtual environment is nearly identical to my experience in a real environment. |  |
|  | 3. The feeling of my movement in the virtual environment is very real.  4. I am completely immersed in the virtual environment |  |
| Perceived enjoyment | 1. I find learning in virtual reality to be fun. | Tokel and Isler (2015) |
|  | 2. Learning using virtual displays is a joy. |  |
|  | 3. I enjoy using virtual reality to learn. |  |
| Learning satisfaction | 1. I am very satisfied with this virtual reality/computer-based learning experience. | Lee et al. (2010) |
|  | 2. A wide variety of learning materials are provided in this virtual reality/computer-based learning environment. |  |
|  | 3. I am satisfied with the instant information I receive in this virtual reality/computer-based learning environment. |  |
|  | 4. I am satisfied with this virtual reality/computer-based learning environment. |  |
|  | 5. I am satisfied with the overall learning effect. |  |
| Perceived learning effect | 1. I learned a great deal concerning this topic. | Lee et al. (2010) |
|  | 2. I have a good understanding of the basic concepts associated with these materials. |  |
|  | 3. I learned to identify important and major issues regarding this topic. |  |

1. **Appendix B**

| The Seven Major Areas of Knowledge in the Pandemic Science Museum |
| --- |

1. What is the novel coronavirus? For example, the novel coronavirus 3D model, transmission route, and on-the-spot performance.

2. Knowledge of work area protection. This category includes entering the building to work, entering the room to work, dining in the cafeteria, commuting to and from work, and disposing of discarded masks.

3. Characteristics of the novel coronavirus. For example, what is the difference between the novel coronavirus and the SARS virus or MERS virus? What are the 7 differences between the novel coronavirus and the influenza virus?

4. Personal protection. For example, how should a mask be chosen? How do special people wear masks? What is a close contact? What are precautions for those who experience close contact?

5. Knowledge of home protection. For example, how to ensure home protection, what protection procedures exist, and what precautions exist.

6. The procedure of medical treatment. When should medical advice be sought? Precautions for medical treatment. How can pneumonia cases infected by COVID-19 be identified in the clinic? Prevention and control measures for pneumonia caused by the novel coronavirus.

7. How much do you know about the COVID-19 vaccine? For example, vaccine classification related to the novel coronavirus and novel coronavirus vaccine-related knowledge.
